# Image-guided study of swine anatomy as a tool for urologic surgery research and training^1^

**DOI:** 10.1590/ACB351208

**Published:** 2021-01-20

**Authors:** Jacob Hindrik Antunes Smit, Eduardo Piotto Leonardi, Rosa Helena de Figueiredo Chaves, Ismari Perini Furlaneto, Cezar Massoud Salame da Silva, Simone de Campos Vieira Abib, Adenauer Marinho de Oliveira Góes

**Affiliations:** I Graduate student, School of Medicine, Centro Universitário do Estado do Pará, Belém-PA, Brazil.; II MSc, Associate Professor, Department of Urology, School of Medicine, Centro Universitário do Estado do Pará, Belém-PA, Brazil.; III PhD, Grupo de Pesquisa Experimental, Centro Universitário do Estado do Pará, Belém-PA, Brazil.; IV PhD, Grupo de Pesquisa Experimental, Centro Universitário do Estado do Pará, Belém-PA, Brazil.; V MSc, Associate Professor, Department of Radiology, School of Medicine, Centro Universitário do Estado do Pará, Belém-PA, Brazil.; VI PhD, Associate Professor, Department of Surgery, Universidade Federal de São Paulo-SP, Brazil.; VII PhD, Full Professor, Department of Vascular Surgery, Grupo de Pesquisa Experimental, Centro Universitário do Estado do Pará, Belém-PA, Brazil.

**Keywords:** Urologic Surgical Procedures, Tomography, X-Ray Computed, Anatomy, Swine

## Abstract

**Purpose:**

To describe the anatomy of the swine urinary system using computed tomography and to discuss the role of this animal as an experimental model for urological procedures.

**Methods:**

Three male Landrace pigs underwent computed tomography and the anatomy of the urinary system and renal circulation was analyzed and described.

**Results:**

In all animals, 2 kidneys, 2 ureters and one bladder were identified. Each kidney presented a single renal artery vascularization, with a mean diameter on the right of 4.45 and 5.31 mm on the left (p < 0.0001) and single renal vein drainage, with a mean diameter on the right of 5.78 and 5.82 mm on the left (p = 0.0336). The average renal length was 9.85 cm on the right and 10.30 cm on the left (p < 0.0001). The average renal volume was 113.70 cm^3^ on the right and 109.70 cm^3^ on the left (p < 0.0001). The average length of the ureter was 19.78 cm on the right and 22.08 cm on the left (p < 0.0001). The average bladder volume was 423.70 cm^3^.

**Conclusions:**

The data obtained show similarities with human anatomy, suggesting the viability of the swine model for planning preclinical trials, basic research, refinement in experimental surgery and surgical training for urological procedures.

## Introduction

Animal models are widely used in experimental research with rats being the main species used in basic research; however, pigs play a prominent role in studies of more complex surgical techniques^1^^,^ 2.

Anatomical and physiological similarities of various systems, such as the urinary and circulatory, that exist between pigs and humans, combined with the wide availability and reasonable prices, make these animals a good option for research models and training in surgery^3^–^7^.

Pigs are used as an experimental model for urological and endourological procedures, such as percutaneous nephrolithotomy^8^^,^ 9, percutaneous renal access^10^, ureterocalicostomy (open and laparoscopic)^11^ and kidney transplantation^3^^,^ 12.

The literature presents anatomical descriptions of the swine urinary system, addressing the pyelocaliceal system^7^^,^ 13, extra and intrarenal vascularization^4^^,^ 5, the ureters^14^ and the urethra^15^, as well as studies of comparative anatomy. Most of these descriptions were based on surgical dissection, plastination and invasive exams, such as angiography, with a few studies using more modern noninvasive imaging methods, like multislice computed tomography (CT). Thus, this research aimed to describe the porcine urinary system based on computed tomography images and to discuss similarities and differences to the human anatomy, as well as, based on tomographic findings, to discuss the application of the porcine model in surgical training and research regarding the urinary system.

## Methods

The study was approved by the institution’s ethics committee on the use of animals (CEUA CESUPA 01/2017).

Three Landrace male pigs, weighing 45.4, 49.2 and 52.3 kg, were used for CT imaging. The animals were kept in a standard environment with adequate housing conditions (temperature and humidity control) and fasted for 12 h before the exam.

### Anesthetic protocol

The CT scans were performed under general anesthesia and monitoring, performed by a veterinarian. As pre-anesthetic medication, a combination of ketamine hydrochloride (15 mg/kg) and xylazine hydrochloride (1.5 mg/kg) was administered by the intramuscular injection.

Then the marginal vein of the ear was accessed with a peripheral catheter (22G), through which hydration with a physiological solution and anesthetic induction, using propofol at a dose of 2.5–5 mg/kg, were instituted. The maintenance was performed with the same agent through continuous infusion (0.1–0.2 mg/kg/min). After being in the anesthetic plane, the swine was positioned in ventral decubitus for CT image acquisition.

All animals maintained spontaneous respiration, with no need of intubation.

### Computed tomography

The CT scans were performed on a 64-channel tomography with 0.625 mm cuts. The intravenous iodinated contrast used was iohexol (120 mL at a flow rate of 5 mL/s).

### Image analysis

Images were evaluated through the software Horos v3.1.0 (Horosproject.org).

For anatomical descriptions, the terms “cranial” and “caudal” were adopted as corresponding to “superior” and “inferior” in humans, respectively.

The following parameters were measured:

Vascular parameters

- Renal artery: diameter of the proximal segment.
- Left renal vein: diameter at the renal hilum and at the point of maximum narrowing between the aorta and the anterior mesenteric artery.
- Right renal vein: diameter at the renal hilum.

Arterial branching pattern was classified accordingly to the types proposed by Evan *et al*.^16^.

Urinary parameters

**Figure 1:**
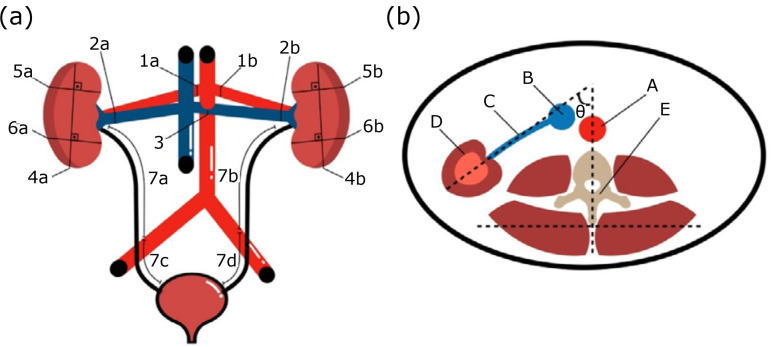
Anatomical landmarks used for measurement. **(a)** Vascular diameters: right renal artery (1a), left renal artery (1b), right renal vein (2a), left renal vein on the renal hilum (2b), left renal vein on the maximum point of compression (between the aorta and the anterior mesenteric artery) (3). Urinary measurements: the length between the most cranial and caudal point of the right and left kidneys (4a/4b), the width of the right and left cranial renal poles (5a/5b) and the width of the right and left caudal renal poles (6a/6b), the length of the right and left abdominal ureteral segments (7a/7b), and the right and left pelvic ureteral segments (7c/7d). **(b)** Angle of the renal hilum: an imaginary line drawn in the center of the vertebral body (E); a virtual line drawn on the path of the renal vein (C) from the kidney (D) to the caudal vena cava (B). abdominal aorta (A).

- Renal length: the greater distance between the cranial and caudal edges.
- Cranial and caudal pole width: from an axis, perpendicular to the length, drawn in the widest segment of the renal poles (cranial and caudal).
- Kidney hilum angle: determined by the technique proposed by Sakate *et al*.^17^, comparing the renal hilum pathway to a line drawn by the vertebral body, as shown in Fig. 1.
- Ureteral length: from the ureteropelvic junction to the ureterovesical junction. The external iliac artery was used as a reference point to divide the ureter into abdominal (cranial) and pelvic (caudal) ureteral segments.
- Kidney and bladder volumes: measured using the ROI (region of interest) tool from Horos v3.3.0 software.

The landmarks used to measure the anatomical features can be visualized in Fig. 1.

### Statistical analysis

The analyses were performed using the software’s GraphPad Prism v8.4.3 and Bioestat v5.3, considering significant values of p ≤ 0.05.

From the distributions of n = 3 observations, an empirical data distribution with 500 observations was independently calculated for each data set using the computational method of data generation (Bioestat) and then, the normality of these distributions was verified using the Shapiro-Wilk test. The mean, standard deviation and coefficient of variation were determined for all data sets and, considering that the distributions were estimated independently, the comparison between them was made by the Mann-Whitney test.

## Results

**Figure 2:**
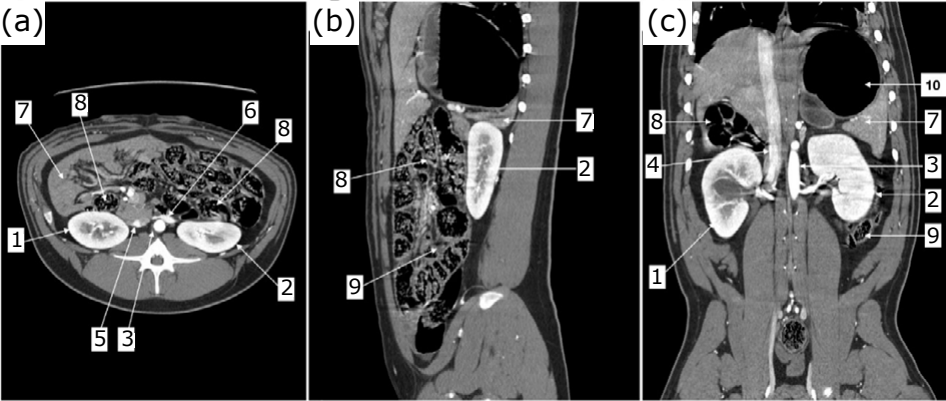
Computed tomography cuts with intravenous contrast in animal 1. **(a)** transversal section; **(b)** sagittal section; **(c)** coronal section; 1: right kidney; 2: left kidney; 3: abdominal aorta; 4: caudal vena cava; 5: right renal vein; 6: left renal vein; 7: liver; 8: small intestine; 9: large intestine; 10: stomach.

Anatomical relations are observed in Fig. 2. In all animals, 2 kidneys, 2 ureters and one bladder were identified. Animal 1 presented a simple renal cyst on the right side.

**Figure 3:**
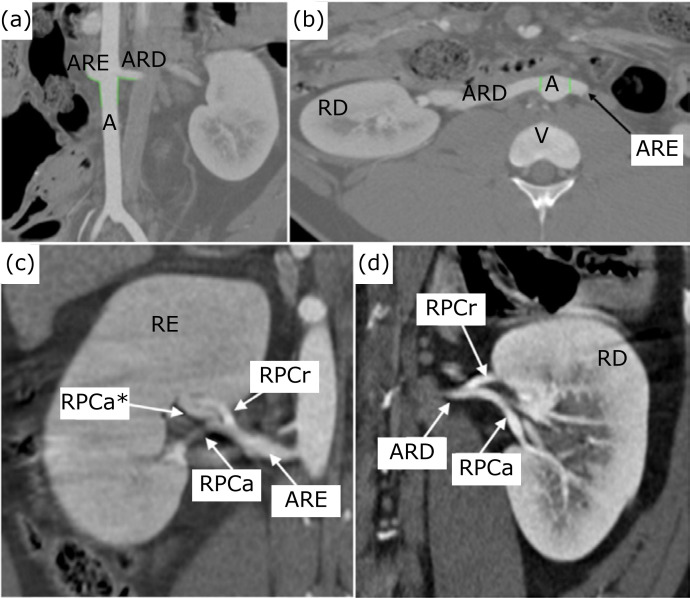
Computed tomography cuts with intravenous contrast. **(a)** coronal section in posteroanterior view; **(b)** axial section; **(c)** and **(d)** coronal sections in posteroanterior view (branching pattern of the renal artery, type II and Ia respectively). A: aorta; ARE: left renal artery; ARD: right renal artery; RD: right kidney; RE: left kidney; V: vertebra; RPCr: cranial pole branch; RPCa: caudal pole branch; RPCa*: vessel emitted from the caudal pole branch to the cranial pole.

Each kidney showed a vascularization by a single renal artery emerging from the abdominal aorta near the 2nd lumbar vertebra, symmetrically (Fig. 3a); except in animal 1, which had an emission of the right renal artery 8.5 mm more caudal than the left renal artery. No extra hilar arterial branches were identified. Next to the abdominal aorta, the diameter of the right renal artery varied between 3.50 and 5.14 mm with a mean of 4.45 mm and between 5.08 and 5.70 mm with a mean of 5.31 mm on the left. The mean diameter was statistically superior on the left renal artery (p < 0.0001) (Fig. 3b).

The images obtained allowed the identification of the arterial branching pattern in all kidneys, except for the right kidney of animal 1. Based on the classification of Evan *et al*.^16^, the animals 2 and 3 presented cranial and caudal polar branches with a distal bifurcation into anterior and posterior branches, characterized as type Ia (Fig. 3d). In the left kidney of animal 1, a short cranial polar branch with anterior and posterior branching at the renal artery topography was noticed, associated with the emission of a vessel from the caudal polar branch towards the cranial pole (type II) (Fig. 3c).

**Figure 4:**
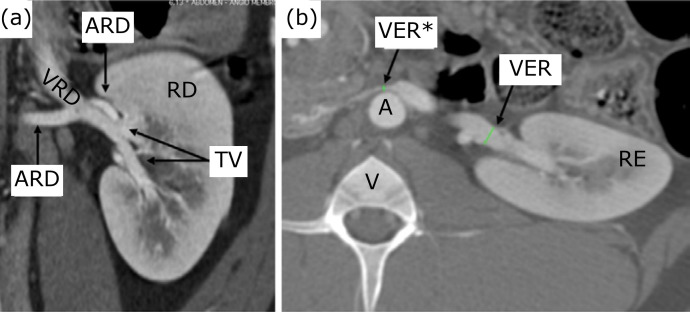
**(a)** coronal section in posteroanterior view; **(b)** axial section; RD: right kidney; ARD: right renal artery; TV: venous tributaries; VRE: left renal vein; VRE*: left renal vein at the maximum point of compression.

Two or three venous tributaries converged to form a single renal vein on each side (Fig. 4A), which flowed into the caudal vena cava between the last thoracic vertebra and the 2nd lumbar vertebra. Near the renal hilum, the diameter of the renal vein varied between 4.90 and 6.40 mm on the right, with an average of 5.78 mm and between 5.26 and 6.35 mm on the left, with an average of 5.82 mm. The venous diameter measured at the hilum was statistically superior on the left side (p = 0.0336). A narrowing of the left renal vein, between the aorta and the anterior mesenteric artery (analogous to the nutcracker syndrome in humans^18^), was observed in all animals (Fig. 4b). At this point, the renal vein diameter average was 2.78 mm.

A renal cyst equivalent to a Bosniak^19^ lesion was identified in the right kidney of animal 1.

**Figure 5:**
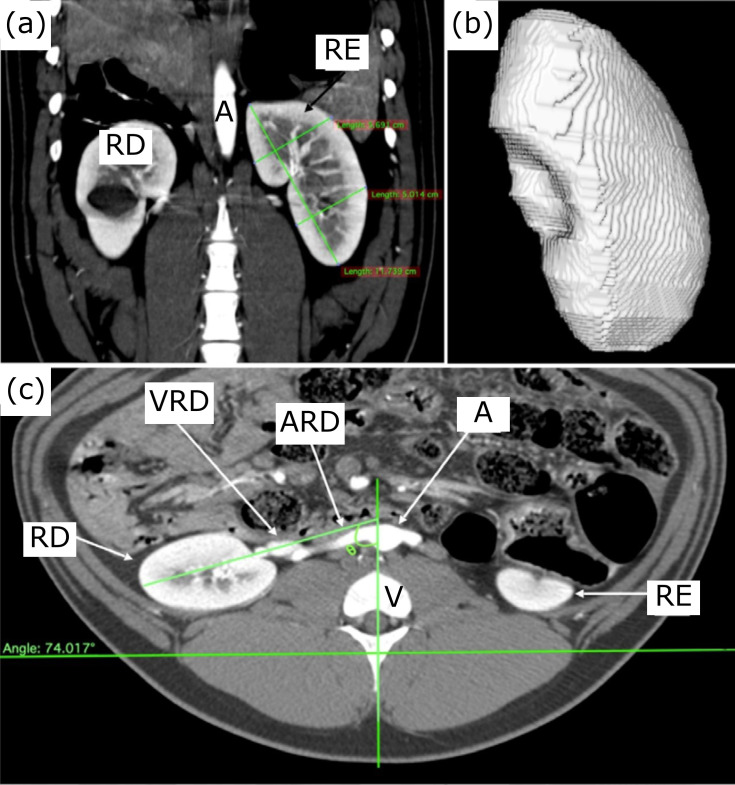
**(a)** coronal section in anteroposterior view; **(b)** 3D reconstruction of the right kidney;**(c)**: axial section; RE: left kidney; RD: right kidney; A: aorta; V: vertebra; VRD: left renal vein; ARD: right renal artery.

The renal length varied from 9.10 to 10.46 cm on the right, with an average of 9.85 cm, and from 9.22 to 11.70 cm on the left, with an average of 10.30 cm. The renal length was statistically superior on the left side (p < 0.0001). The width of the cranial renal pole varied between 5.11 and 6.45 cm, with an average of 5.82 cm on the right, and between 5.65 and 6.57 cm, with an average of 6.05 cm on the left. The width of the cranial pole was statistically superior on the right side (p < 0.0001). The width of the caudal renal pole varied between 4.29 and 5.52 cm, with an average of 4.92 cm on the right, and between 4.48 and 5.16 cm, with an average of 4.85 cm on the left, with no statistical difference between the sides (p = 0.0625) (Fig. 5a). The renal volume varied between 79.78 and 130.50 cm^3^ on the right, with an average of 113.70 cm^3^, and between 78.32 and 125.50 cm^3^ on the left, with an average of 109.70 cm^3^. The renal volume was statistically higher on the right side (p < 0.0001) (Fig. 5b).

The angle of the renal hilum varied between 73.53 and 74.85° on the right, with an average of 74.08°, and between 63.87 and 70.39° on the left, with an average of 67.59°. The right renal hilum angulation was statistically superior when compared to the left side (p < 0.0001) (Fig. 5c).

**Figure 6:**
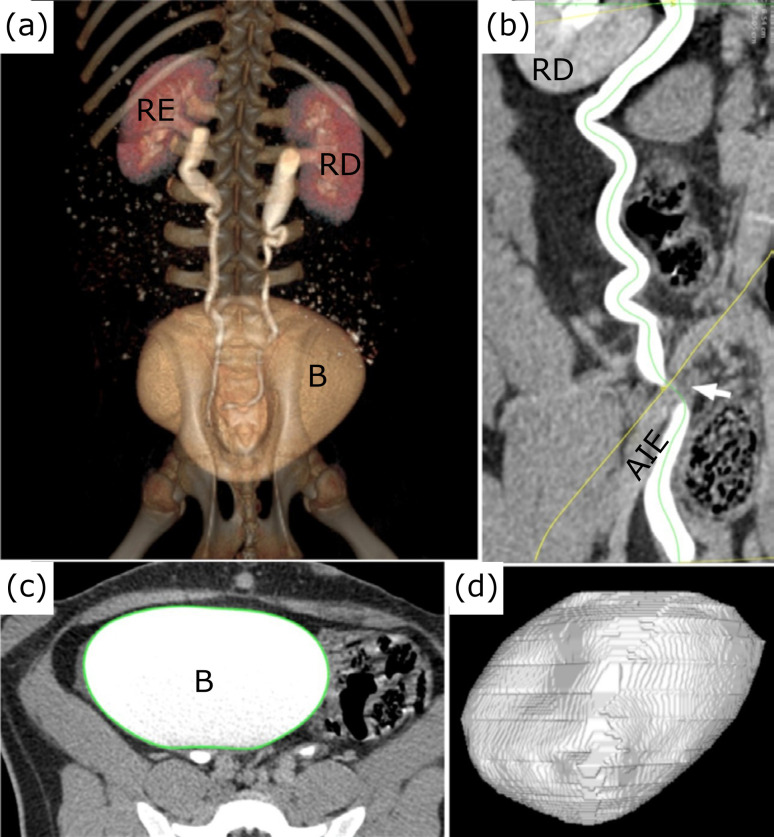
**(a)** maximum intensity projection image (MIP); **(b)** ureteral length obtained through the “curved-multiplanar reformation” tool; **(c)** axial cut (to measure bladder volume); **(d)** 3D bladder reconstruction. White arrow: ureteral narrowing point over the external iliac artery; IEA: external iliac artery; RD: right kidney; RE: left kidney; B: bladder.

The ureters presented a slight tortuosity along their path. The total length varied between 16.06 and 23.13 cm on the right, with an average of 19.78 cm, and between 21.55 and 23.21 cm on the left, with an average of 22.08 cm, the total ureteral length was statistically superior on the left (p < 0.0001). On the right side, the abdominal segment of the ureter varied between 12.18 and 15.85 cm (mean of 13.55 cm) and between 13.41 and 17.52 cm on the left (mean of 15.75 cm), and the length of the abdominal segment was statistically superior to the left (p < 0.0001). The pelvic segment of the ureter measured between 3.88 and 7.28 cm on the right (mean of 6.16 cm) and between 5.61 and 8.22 cm on the left (mean of 6.42 cm), but no statistical difference was identified between them (p = 0.9302). A ureteral narrowing was identified when crossing the external iliac artery in all animals. Only in pigs 1 and 3 a narrowing at the vesicoureteral junction was detected, both on the left side (Figs. 6a and b).

The bilateral ureteral implantation was observed at the posterior wall of the bladder, except in animal 1, in which occurred at the posterolateral bladder wall for the left ureter. The bladder shape was similar to humans, with volume ranging from 322 to 621 cm^3^ and a mean of 423.70 cm^3^ (Figs. 6c and d).

Tables 1 to 4 present the anatomical parameters analyzed in this study.

## Variation coefficients

The highest variation coefficients were found in the measurements of right renal volume (29.60%), followed by bladder volume (28.94%), left renal vein diameter at the point of compression (23.90%), lengths of pelvic ureteral segments (23.82% on the right and 18.33% on the left), renal volumes (18.43% on the right and 18.23% on the left) and the width of the right caudal pole (17.73%).

**Table 1:** Vascular measurements.

Measure (mm)		Minimum		Maximum		Median		Mean		Standard deviation		CV%		p-value* (right vs. left)
Right renal artery diameter		3.50		5.14		4.45		4.45		0.65		14.50		< 0.0001†
Left renal artery diameter		5.08		5.70		5.10		5.31		0.30		5.50		
Right renal vein diameter		4.90		6.40		5.78		5.78		0.67		11.60		0.0336†
Left renal vein diameter		5.26		6.35		6.10		5.82		0.45		7.80		
Left renal vein diameter (compression point)		1.83		3.35		3.12		2.78		0.66		23.90		Not applicable

* Mann-Whitney text.

† Statistically significant. CV: Coefficient of variation. Source: Compiled by authors, 2019.

**Table 2:** Urinary measurements.

Measure (mm)		Minimum		Maximum		Median		Mean		Standard deviation		CV%		p-value* (right vs. left)
Total length of the right ureter		16.06		23.13		19.20		19.78		2.83		14.30		< 0.0001†
Total length of the left ureter		21.55		23.21		21.63		22.08		0.74		3.36		
Length of right ureter abdominal segment		12.18		15.85		12.31		13.55		1.74		12.84		< 0.0001†
Length of left ureter abdominal segment		13.41		17.52		15.94		15.75		1.69		10.71		
Length of right ureter pelvic segment		3.88		7.28		6.89		6.16		1.47		23.82		0.9302
Length of left ureter pelvic segment		5.61		8.22		5.69		6.42		1.18		18.33		
Right kidney length		9.10		10.46		9.93		9.85		0.57		5.77		< 0.0001†
Left kidney length		9.22		11.70		10.25		10.30		9.90		9.60		
Right cranial pole width		5.11		6.45		6.01		5.82		0.57		9.80		< 0.0001†
Left cranial pole width		5.65		6.57		5.88		6.05		0.40		6.70		
Right caudal pole width		4.29		5.52		4.87		4.92		0.49		9.95		0.0625
Left caudal pole width		4.48		5.16		4.95		4.85		0.29		6.00		

* Mann-Whitney test.

† Statistically significant. CV: Coefficient of variation. Source: Compiled by authors, 2019.

**Table 3:** Renal hilum angle measurements.

Measure (degrees)		Minimum		Maximum		Median		Mean		Standard deviation		CV%		p-value* (right vs. left)
Right renal hilum angle		73.53		74.85		74.01		74.08		0.54		0.73		< 0.0001†
Left renal hilum angle		63.87		70.39		68.82		67.59		2.88		4.27		

* Mann-Whitney test.

† Statistically significant. CV: Coefficient of variation. Source: Compiled by authors, 2019.

**Table 4:** Volume measurements.

Measure (cm^3^)		Minimum		Maximum		Median		Mean		Standard deviation		CV%		p-value* (right vs. left)
Right kidney volume		79.78		130.50		122.00		113.70		20.96		18.43		<0.0001†
Left kidney volume		78.32		125.50		118.70		109.70		20.00		18.23		
Bladder volume		322.00		621.00		380.0		423.70		122.60		28.94		Not applicable

* Mann-Whitney test.

† Statistically significant. CV: Coefficient of variation. Source: Compiled by authors, 2019.

## Discussion

Anatomical knowledge is paramount for developing experimental and training models. However, although swine are already consolidated models for these purposes, there are only few theoretical references about radiological methods for anatomical descriptions of the urinary system.

Experimental research using the porcine model has been published in the field of renal transplantation^3^, percutaneous nephrolitotripsy^20^, ureterocystostomy^21^, ureterorenoscopy and laparoscopic pyeloplasty^2^. Although different breeds are used in urological research, the review of the available literature suggests that pigs classified as “minipigs”, such as Yucatan, Hanford, Göttingen and Sinclair, are not usually chosen for urological studies.

Yorkshire pigs were used in the improvement of techniques, such as robot-assisted anatrophic nephrolithotomy, reducing the morbidity during this procedure^22^. Animals of the same breed, with an average weight of 35 kg, were used to evaluate the efficiency of a new retroperitoneal dialysis technique^23^ and animals between 3 and 4 months (weighting between 29 and 35 kg) were used in experiments of ischemia and renal reperfusion^24^. The anatomy of the renal arteries, including their angles and branching patterns, was described by angiography in Landrace and Yorkshire pigs weighing between 50 and70 kg^25^.

Landrace pigs weighting between 30 and 40 kg, the same breed used in this study, took part in research about ischemia, reperfusion and renal transplantation^26^^,^ 27. In another study, 3-month-old pigs (weighting about 40 kg) were used to evaluate the efficacy of arterial clamping during the treatment of renal tumors by cryoablation technique^28^. This breed represents a good option for anatomical studies of the urinary system because it is available at an affordable cost, besides being widely used in research in the urological and endourological fields.

As well as being considered a precise and noninvasive method, CT helps to clarify the information provided by other exams, such as ultrasonography, and enhances anatomical findings. It became an important tool for urological preoperative planning^29^. However, information regarding the description of swine urological anatomy by CT is still limited.

### Swine anatomy × human

#### Renal vascularization

The results showed that, as in humans, swine renal vascularization is provided by a single renal artery^4^. Although anatomical variations may occur from 30 to 40% in humans, being the accessory polar renal artery the most frequently observed^30^, such variations are rarely observed in pigs (1.7%)^4^ and were not detected in this study.

The most common branching pattern of the renal artery in humans is the division into two polar arteries: one cranial and one caudal, each one giving two branches, one anterior and other posterior; however, variations are frequently reported^31^^,^ 32. In pigs, the branching pattern of the renal artery was classified by Evan *et al*.^16^ in the following types: pattern Ia, with the division of the renal artery into cranial and caudal polar branches, which branch into anterior and posterior segmental branches; pattern Ib, resembles Ia, but has a short cranial polar branch, with the emission of segmental branches directly from the renal artery; pattern II, similar to Ib, but the caudal polar branch gives a branch that irrigates the cranial pole; in pattern III there are multiple renal arteries. The most frequent pattern, as in this study, is the Ia, detected in 77–97%^4^^,^ 16.

In both pigs and humans, renal arteries often originate from the abdominal aorta between the first and second lumbar vertebrae (L1-L2)^29^^,^ 33 ^,^ 34. In pigs, the numbering of the vertebrae may vary, as different breeds may differ in the number of vertebrae^35^. The asymmetry in the level of origin of these arteries is described in pigs and humans^4^^,^ 5. In this study, only one animal presented such asymmetry, being the origin of the right renal artery 8.5 mm caudal to the left renal artery. This asymmetry, although small, was greater than previously reported in pigs (4.48 mm) of different races (between 85–90 kg) and humans (2.9 mm)^4^.

The renal artery diameter in adult humans varies between 4.0 and 5.9 mm and between 5.1 and 5.4 mm in pigs^4^^,^ 25 ^,^ 34 ^,^ 36. In pigs, this data was collected using surgical dissection, plastination and angiography in animals of different breeds between 51 and90 kg^4^^,^ 25. In contrast to the literature, the present study showed a mean diameter of 4.45 mm on the right and 5.31 mm on the left, being the left renal artery diameter statistically larger than the right renal artery. For procedures in which the diameter of these vessels is an important factor, it is known that the weight of the pig has a small influence since, in these animals, the correlation between the caliber of the renal arteries and weight is disproportional, meaning there are minimal variations in the diameter between pigs of different weights^25^.

The renal venous drainage of the pig is also similar to humans. A single renal vein is the most observed pattern in both species^5^, as in the animals studied. Venous variations were rarely found in pigs, being reported additional renal veins in 2.13% after the analysis of 94 kidneys^37^. In humans, renal venous anomalies are more frequent, being the multiplicity of renal veins (15–30%) more common on the right side and, less frequently (2–17%), circumaortic and retroaortic renal veins on the left side^38^. The formation of renal veins, in humans and pigs, often occurs by the confluence of cranial and caudal venous tributaries, at the upper margin of second lumbar vertebrae. The number of tributaries varies between 2 and 4 in pigs and 2 and 6 in humans^37^.

According to the literature, in humans, the diameter of renal veins may vary between 5 and 17 mm^37^. A tomography study points out an average diameter of 6.98 mm at the right side and 6.36 mm at the left side in men and 6.69 mm at the right and 6.04 mm at the left side in women^34^. In Great Polish White pigs, between 70 and 110 kg, the average previously described is 10.94 mm, with variations between 4.5–15.8 mm^37^. In this study, an average of 5.78 mm was found on the right side and 5.82 mm on the left side, with variations between 4.90–6.40 mm and 5.26–6.35 mm, respectively. The caliber of the left renal vein measured at the renal hilum was statistically superior when compared to the right renal vein with the same topography. The disparity between this study and previous literature results may be related to differences regarding animal breeds and measurement techniques.

In humans, the nutcracker syndrome, which is characterized by the compression on the left renal vein between the aorta and the superior mesenteric artery, may cause lumbar pain, hematuria and proteinuria^39^. There was no previous report of a similar situation in pigs. This group, during the description of the swine vascular anatomy by CT angiography, in another project, detected this compressive phenomenon (data not yet published) and, in another study, conducted in humans, this compression was observed in 24.4% of adults submitted to CT examinations^40^. The three evaluated animals presented, on average, a 51% diameter reduction of the left renal vein at the angle between the aorta and the anterior mesenteric artery (analogous to the human superior mesenteric artery).

#### Kidney

The morphometric similarities between human and swine kidneys have been widely described^4^^,^ 5 ^,^ 7. In adult humans, the average kidney length varies between 10.5 and 11.1 cm and discrete differences may occur in the sides^41^^,^ 42. In this study, the mean renal length was 9.85 cm on the right and 10.30 cm on the left; these dimensions were close to those reported for humans by Gómez *et al*.^7^ and Arenas *et al*.^5^ who, after surgical dissection in pigs of various breeds weighing between 85 and 95 kg, identified an average length of 12 cm. This discrete difference compared to the results obtained may be related to the size of the animals studied and the method used for measuring^41^.

Both pigs and humans have a larger and more medial and cranial located renal pole in comparison to the caudal pole. In the animals studied, an average cranial pole width of 5.82 cm on the right and 6.05 cm on the left was found, and 4.92 cm on the right and 4.85 cm on the left for the caudal pole. These measurements are similar to those described by Gómez *et al*.^7^, who identified the average cranial pole width of 5.66 cm on the right and 5.72 cm on the left and caudal pole width of 5.26 cm on the right and 5.21 cm on the left. The average dimensions in humans are 6.44 and 5.49 cm for the cranial and caudal poles, respectively. Certainly, these similarities between the species are relevant when selecting the swine model for procedures in which renal dimensions are an important issue.

The average renal volume in humans is 94.16 cm^3^ on the right and 98.07 cm^3^ on the left and, in younger and heavier male patients, there is a tendency of larger renal volumes, especially on the left side^43^. Swine’s kidney volume is influenced by weight, gender, age and pathologies, such as the cystic kidney disease^44^. No references were detected among pigs with weights similar to those used in this study, so a comparison could not be established. Between the surveyed pigs, the mean kidney volumes were 113.70 cm^3^ on the right and 109.70 cm^3^ on the left.

No references describing the swine hilar kidney angle were found. According to the available literature, the mean right hilar angle in humans varies between 42.47 – 61° and 38.78 – 55° on the left side^17^^,^ 45 ^,^ 46. In the present study, the renal hilar angle average was 74.08° to the right and 67.59° to the left.

Renal rotation anomalies were not reported in pigs. In humans, they may be associated with renal ectopy, which plays an important role in the formation of ureteropelvic obstructions. Among these alterations, the most common are the nonrotations and incomplete rotations^17^. Also, there is a higher incidence of renal lithiasis in patients with an insufficient rotation of the renal hilum, i.e., with a smaller renal hilum angle and pelvis arranged more anteriorly^46^, a fact that may be associated with the low frequency of renal lithiasis in pigs, since they present medially rotated kidneys.

#### Ureters and bladder

Information about these structures in pigs is limited in the literature.

Human ureters cross the common iliac arteries and this vessel is used as a border between the cranial/abdominal and caudal/pelvic portions of the ureters. In pigs, ureters follow a similar path^47^, however, the best nomenclature for the crossed artery would be “external iliac artery”, since the aorta does not bifurcate into common iliac arteries, like in humans, but instead trifurcate into two external iliac arteries and an internal iliac trunk, from which both internal iliac arteries arise.

The ureteral dimensions in humans have been studied by several authors^47^–^51^ who describe a ureteral length ranging from 25 to 30 cm, larger when compared to the evaluated swine in this research, with a length of 19.78 cm on the right and 22.08 cm on the left.

In this study, pigs presented larger length measurements on the left side, possibly justified by the higher disposition of the left kidney compared to the right side. A review of the literature found no references about the proportion of the abdominal and pelvic segments of ureters in pigs. In humans, similar lengths were reported between these segments of ureter^48^, but this work demonstrated that, in pigs, the division of the ureter is uneven, with the abdominal segment corresponding to approximately 2/3 of the length.

The human ureter has three points of physiological narrowing: the ureteropelvic junction, the crossing of the common iliac vessels and the ureterovesical junction^48^^,^ 52. The methodology used in this study allowed to verify the narrowing points of the ureteral lumen when crossing the iliac vessels in all animals and at the ureterovesical junction in two animals, both on the left. No narrowing was detected in the ureteropelvic junctions. In humans, the correlation between the points of ureteral narrowing and calculus impaction is well described^50^^,^ 53–55, and, likewise, the swine model can be useful for experiments and training related to lithiasis, although previous description of these narrowing has not been detected in the literature.

No previous descriptions of the mean bladder volume in pigs have been located and, certainly, such volume may vary according to the degree of bladder repletion and the method of measurement. In humans, this mean volume varies between 400 and 600 mL^56^ and, therefore, despite possible measurement biases, it is similar to that found in the evaluated swine, with an average volume of 423.7 mL. Bladder manipulation during urological procedures should be careful due to the fragility of the bladder wall, which is thinner when compared to humans or other mammals^57^.

The urethral anatomy in male pigs makes it difficult to use this model in urological procedures involving the penis and urethra. The corkscrew shape of the tip of the penis makes it difficult to insert a urinary catheter via the urethra^57^. Suprapubic access with catheter placement is an alternative used for this model^58^. Large White, Landrace, Yorkshire and Duroc females, weighing 30–40 kg, were successfully submitted to ureteral catheterization, although difficulty was reported during the procedure^59^^,^ 60.

Among the limitations of this research is the fact that only three animals were studied, all of the same sex, and that the definition and heterogeneity of the images did not allow the measurement of the cortical thickness, the evaluation of the renal pelvis and the description of the pyelocaliceal system.

## Conclusions

Based on the obtained results and comparisons made to the human anatomy, it was found that the pig is particularly useful for the following models of procedures related to the urinary system: total and partial nephrectomy, intrarenal procedures (such as biopsy and nephrolithotomy), kidney transplantation and nephrolithotripsy.

By providing these compared anatomic descriptions, the planning of preclinical trials, basic research and improving surgical training using porcine models in urology may be facilitated.
